# Novel bifunctional cap for simultaneous electroencephalography and transcranial electrical stimulation

**DOI:** 10.1038/s41598-018-25562-x

**Published:** 2018-05-08

**Authors:** Sophia Wunder, Alexander Hunold, Patrique Fiedler, Falk Schlegelmilch, Klaus Schellhorn, Jens Haueisen

**Affiliations:** 1 Institute of Biomedical Engineering and Informatics, Technische Universität Ilmenau, 98693 Ilmenau, Germany; 2 neuroConn GmbH, 98693 Ilmenau, Germany; 3 Department of Neurology, Biomagnetic Center, Jena University Hospital, 07747 Jena, Germany

## Abstract

Neuromodulation induced by transcranial electric stimulation (TES) exhibited promising potential for clinical practice. However, the underlying mechanisms remain subject of research. The combination of TES and electroencephalography (EEG) offers great potential for investigating these mechanisms and brain function in general, especially when performed simultaneously. In conventional applications, the combination of EEG and TES suffers from limitations on the electrode level (gel for electrode-skin interface) and the usability level (preparation time, reproducibility of positioning). To overcome these limitations, we designed a bifunctional cap for simultaneous TES–EEG applications. We used novel electrode materials, namely textile stimulation electrodes and dry EEG electrodes integrated in a flexible textile cap. We verified the functionality of this cap by analysing the effect of TES on visual evoked potentials (VEPs). In accordance with previous reports using standard TES, the amplitude of the N75 component was significantly decreased post-stimulation, indicating the feasibility of using this novel flexible cap for simultaneous TES and EEG. Further, we found a significant reduction of the P100 component only during TES, indicating a different brain modulation effect during and after TES. In conclusion, the novel bifunctional cap offers a novel tool for simultaneous TES–EEG applications in clinical research, therapy monitoring and closed-loop stimulation.

## Introduction

Transcranial electric stimulation (TES) is a non-invasive method of neuromodulation, where weak electric currents (typically between 1 and 2 mA) are applied to the human scalp using surface electrodes. Subtypes of TES include transcranial direct current stimulation (tDCS), transcranial alternating current stimulation (tACS) and transcranial random noise stimulation (tRNS). TDCS creates a static electric field, which polarizes the nervous tissue, leading to a modulation of neuronal activity in the cerebral cortex^1^. TACS creates interferences and resonances of cortical circuits and thus can be used to analyse connections between cortical regions or spontaneous brain activity^2^. The application of random electrical oscillations in tRNS increases neural excitability^3^, especially task-related neural activity in high-frequency tRNS^4^ (100–640 Hz).

The characteristics of an induced TES effect depend on technical parameters, such as size and montage of the surface electrodes, current intensity and waveform, stimulation duration and number of sessions, as well as inter-session intervals^5^. Only a fraction of the applied current actually reaches the brain due to shunting effects of superficial tissue layers^6^. Moreover, TES effects depend on electrode montages, where typical electrode sizes of 25–35 cm² allow only for mediocre focality^5^. While TES of a few seconds causes brief, but reversible stimulation effects, at least 10 min of TES are necessary to induce prolonged after-effects, which can last up to one hour^1^.

TES benefits from its simplicity, low costs (approximately 5,000 USD) and the mobility of the equipment, as only electrodes and a stimulator are necessary for application^2^. TES has mild side effects ranging from mild tingling and itching sensation to moderate fatigue and headache^7^. Possible skin redness can be caused by vasodilatation beneath the electrode surface^8^, but there is no evidence of significant temperature rises^9^ or toxic effects resulting from TES. According to a meta-analysis on tDCS safety, protocols within the range of the following parameters are regarded as safe: intensity ≤ 4 mA, duration ≤ 40 min, electric charge ≤ 7.2 C^6^, and current densities^10^ of less than 80 µA/cm². Contraindications include metallic implants in the head, implanted defibrillator and pacemaker, serious brain injury and open skull. Chronic skin disorder, pregnancy and a history of seizure or stroke might also be exclusion criteria^11^. Since tDCS is the most intensively investigated type of TES, recommendations and safety guidelines are the furthest advanced for this technique and can likely be transferred to tACS^12^ and tRNS^3^ as major technical aspects of the application are the same.

TES has been indicated to improve symptoms of psychiatric and neurophysiological diseases^13–15^, and TES has been applied for the therapy of various conditions, including stroke^16–19^ and depression^20–22^. A major open research question concerns the duration of therapeutically lasting effects^23^. Furthermore, TES has been used in neurophysiological research studies investigating cognitive brain functions^24–26^ and sensory processing^27–30^.

Despite its successful application, our understanding of the underlying mechanisms of TES remains limited. Combining TES techniques with electroencephalography (EEG) offers the potential for fundamental new insights in this field, as taking simultaneous measurements with EEG enable the analysis of the direct-effects and after-effects of stimulation^25,31–33^. EEG and TES are well compatible as they possess similar spatial and temporal characteristics: they can record or stimulate brain activity at millisecond scale time resolution and at millimetre to centimetre scale spatial resolution. This compatibility enables monitoring and adjusting TES parameters based on simultaneously recorded EEG parameters, a technique known as closed-loop brain state-dependent non-invasive transcranial brain stimulation^34^.

The first successful simultaneous TES–EEG recording was published by Schroeder and Barr in 2001^31^. Using a 3-stage amplifier system, they were able to analyse quantitative EEG parameters of one bipolar configuration of Ag/AgCl electrodes during TES^31^.

Despite of the progress in EEG amplifier techniques that enable simultaneous TES and EEG recordings, some aspects of combined stimulation and recording, such as electrode positioning and preparation, pose considerable limitations for mobile applications. In conventional TES and TES–EEG experiments, conductive rubber electrodes are applied to the head using elastic bands, while saline-soaked sponges^35–38^ or electrode paste^33^ on the rubber electrodes serve to create the required electrical contact to the skin. These electrodes must be placed manually, and their positions should be determined before each application. In addition to necessitating a trained medical staff, these features are a source of error when applying the same setups to different participants or in several sessions to the same participants. Moreover, considerable operator variability exists. Furthermore, the electrode placement is somewhat instable owing to the elasticity of the bands and the variations in the effective electrode contact area due to head curvature. Similar problems exist in the preparation of conventional EEG electrodes and their placement given that EEG caps exist but they are not practical in combination with conventional TES, because they must be put above the stimulation electrodes and therefore preventing access to the stimulation electrodes. Consequently, the majority of TES–EEG studies separately apply TES electrodes and EEG electrodes using different mounting systems. Recently, Roy *et al*.^39^ and van Schouwenburg *et al*.^40^ proposed combined TES–EEG caps incorporating conventional Ag/AgCl electrodes for both EEG and TES to overcome problems of positioning reproducibility. However, the main drawback of using conventional EEG electrodes for TES consists in the risk of exceeding the current density limit^10^ of 80 µA/cm². An additional drawback related to conventional Ag/AgCl electrodes is the required preparation of the scalp with abrasive gel and the use of conductive paste or gel for the electric interface between electrode and skin, which can cause skin irritations^41^ and hair loss^42–44^.

Consequently, our aim was to develop a cap incorporating both EEG and stimulation electrodes, which does not limit electrode size and position. This bifunctional cap aimed to improve the reproducibility of TES–EEG and to overcome drawbacks regarding preparation time, trained staff involvement, and use of consumables such as electrode paste. Further, we aimed to reach high EEG signal quality, mechanical cap stability and flexibility, and to establish methods of cleaning and disinfection. Consequently, another main purpose was the verification of the cap’s TES and EEG functionality. The proof-of-principle study is based on the reproduction of a well-known stimulation effect based on the EEG as outcome measure. Reviewing several TES–EEG approaches, we chose to replicate the effect of tDCS on the N75 amplitude of the pattern-reversal visual evoked potential (VEP) investigated by Antal and colleagues^36^. The pattern-reversal VEP with its main components N75, P100, and N135 represents a robust and reliable outcome measure for tDCS modulation of neural activity of the striate and extra striate cortex^45^. At the same time, good electrode contact on the occipital area of the head is challenging due to head curvature.

We present the realization of a new TES–EEG tool that complies with the specified requirements by using dry multipin electrodes for EEG recording^46,47^ and textile stimulation electrodes for TES^48^ integrated in a flexible textile cap. We specifically adapted the cap design to the chosen TES–EEG paradigm: stimulation electrodes were placed at positions Cz and Oz and EEG electrodes over the visual cortex surrounding Oz. In the proof-of-principle study, we analysed not only the after-effects, but also direct-effects of tDCS (1 mA, 10 min, cathode at Oz) on the VEPs of ten volunteers. The functionality of the novel cap was verified by a reproduction of the known effect of N75 amplitude reduction after the end of tDCS^36^. The newly found effects of amplitude reduction and latency increase of the P100 component during tDCS support the view that cathodal tDCS decreases excitability of the targeted cortical area. The novel cap benefits from improved reproducibility and stability as well as omission of skin preparation and the use of electrode paste. These extents the range of operation for TES–EEG in clinical research, monitoring, and therapy, e.g. allowing for the first time mobile and home applications.

## Results

### Realization of the bifunctional cap

**Figure 1:**
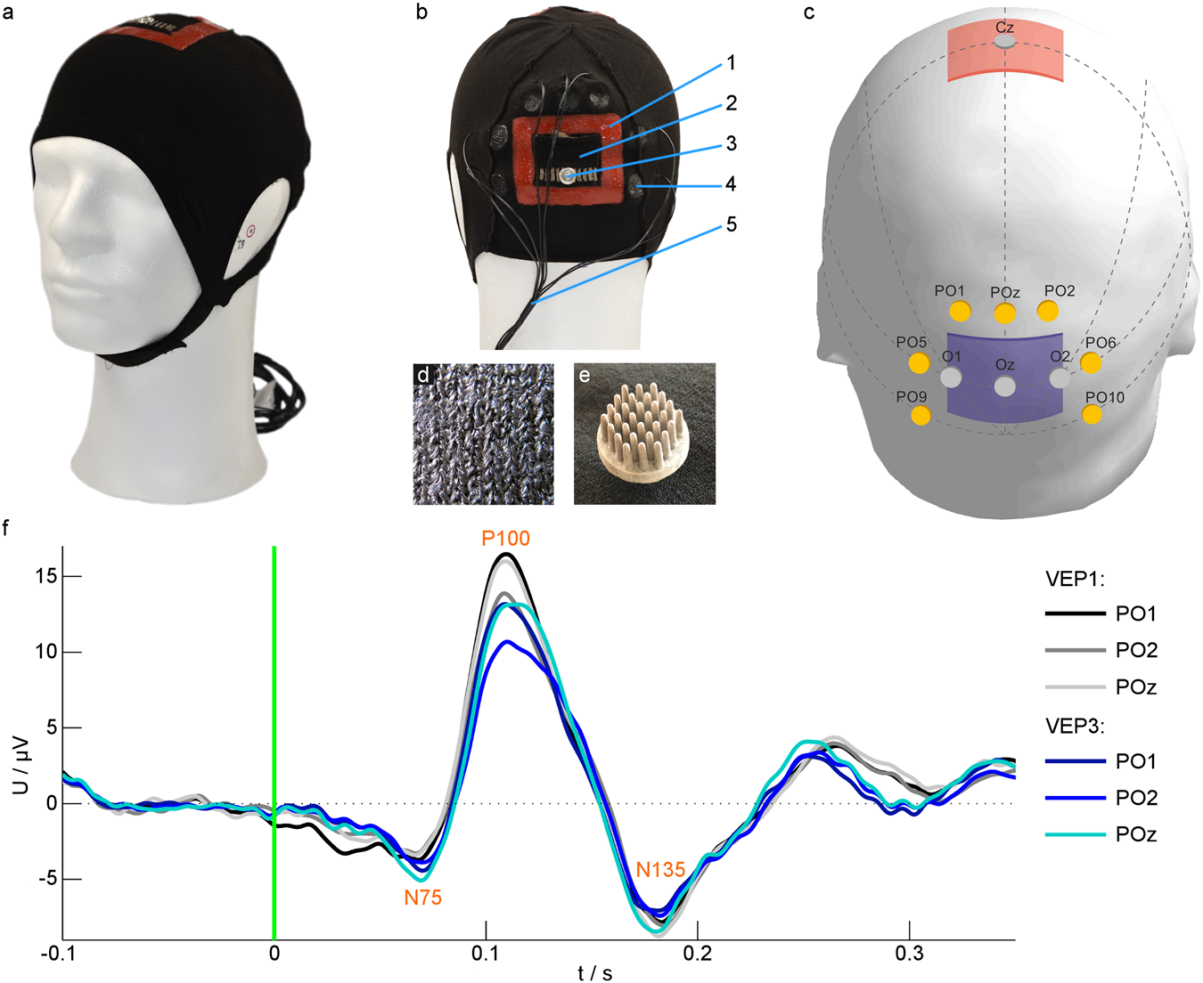
The design of the bifunctional cap was verified by recording VEPs during tDCS. (**a**,**b**) Photos of the cap with cap components: 1 – Silicone frame to prevent electrolyte diffusion; 2 – Textile stimulation electrode; 3 – Snap fastener for electrically connecting the textile stimulation electrode; 4 – Dry EEG electrode; 5 – EEG electrode cables. (**c**) Scheme of the positions of stimulation electrodes: anode (red) over Cz, cathode (blue) over Oz, and EEG recording electrodes (yellow) according to the international 10/10 system^56^. Dotted lines indicate auxiliary lines for electrode positioning. (**d**) The textile stimulation electrode consists of silver-coated threads integrated into the cap fabric. (**e**) Dry polyurethane-based EEG electrodes consisting of 30 pins coated with Ag/AgCl were integrated in the cap. (**f**) Average VEP with the components N75, P100 and N135 of one participant for different channels before (VEP1) and during (VEP3) tDCS. The green line at zero indicates the time of the visual stimulus. | EEG – electroencephalography; tDCS – transcranial direct current stimulation; VEP – visual evoked potential.

The cap resulting from the above outlined requirements is depicted in Fig. 1a,b. We used a flexible textile cap material (cotton, elastane and polyamide) providing maximal elongation (mean ± std) of 234.78% ± 1.40% along and 320.81% ± 1.44% orthogonal to the knitting direction at an applied force of 20 N. Velcro strips underneath the chin ensured stability on the head and sufficient contact pressure at the electrodes. The cap was created by flat knitting and can easily be produced by industrial manufacturing and with production lot sizes of 1 (i.e. customized production). In the stimulation areas, conductive, silver-coated polyamide threads were knitted in the fabric by means of a second knitting magazine, see Fig. 1d, guaranteeing reproducibility of the TES electrode sizes and positions. Pockets were knitted on top of the TES electrodes, which hold saline-soaked sponges to provide an electrolyte reservoir for establishing electrical contact between the stimulation electrode and the scalp. Liquid silicone was inserted into the knitted fabric around the textile electrode to prevent saline diffusion outside the stimulation electrodes. Seven dry multipin electrodes (see Fig. 1e) were integrated in the textile cap around the occipital stimulation area for VEP recording (see Fig. 1c).

The appearance of the cap presented in Fig. 1a–c is due to the paradigm of the chosen verification experiment. The positioning of stimulation and recording electrodes can be customized for any given paradigm.

### Verification of cap design

After the cap realization, we verified design properties like cap fit and influence of laundering on the textile electrodes and the electrode diffusion barrier by experimental procedures.

To test the basic cap fitting characteristic, ten volunteers were fitted with caps and questioned about their wearing perception. All volunteers reported a good and comfortable cap fit during the measurements, which lasted 60 min. TDCS was performed successfully in all volunteers, and no unpleasant skin sensations were reported. The interfacial impedance between the anode and cathode was initially 7.0 ± 4.44 kΩ and decreased during the stimulation.

In an additional volunteer, we verified the cleaning process of the cap, which is important for an anticipated home use. We measured the interface impedances between the electrode and the skin after 50 and 100 laundering cycles. Impedances between an EEG reference electrode on the forehead and the textile cathode were 40.2 ± 3.1 kΩ before washing, 38.5 ± 5.6 kΩ after 50 cycles and 26.7 ± 14.4 kΩ after 100 cycles (see Supplementary Table 1). These impedances decreased by approximately 40% over the measurement duration of 30 min, suggesting that the skin-electrode interface stabilized over the course of stimulation.

High effectiveness of the silicone frame surrounding the stimulation electrodes to prevent electrolyte solution diffusion was observed in ten volunteers during the TES–EEG study. In addition, in one volunteer, we tested the effectiveness of the diffusion barrier after 50 and 100 laundering cycles by means of impedance measurements to verify that laundering does not disrupt the integrity of the barrier. Impedances were measured between the EEG reference electrode on the forehead and four textile positions 5 mm outside the silicone frame (on each side of the frame). In 2457 out of 3024 samples of the impedance measurements, the measurement device’s maximum limit (2,000 kΩ) was exceeded. The remaining samples exhibited an impedance of 466 ± 434 kΩ before washing, 510 ± 337 kΩ after 50 cycles and 408 ± 308 kΩ after 100 cycles (see Supplementary Table 1). None of these impedances exhibited a decreasing trend over the 30-min impedance measurement period. This suggests that the silicone frame successfully prevented diffusion of the saline solution.

Taken together, these results demonstrate successful verification of the cap design including adequate and consistent electrode-skin interface impedance for the textile stimulation electrodes and the reliability of the silicone frame as diffusion barrier.

### After-effects of tDCS

To verify both functional aspects (TES and EEG) of the bifunctional cap, we aimed to reproduce a well-known TES effect on the human EEG. As the chosen paradigm is based on the after-effects of tDCS, we analysed the after-effects of tDCS at first. Thereafter, we present the direct-effects of tDCS since the bifunctional cap enables EEG recording during TES.

**Figure 2:**
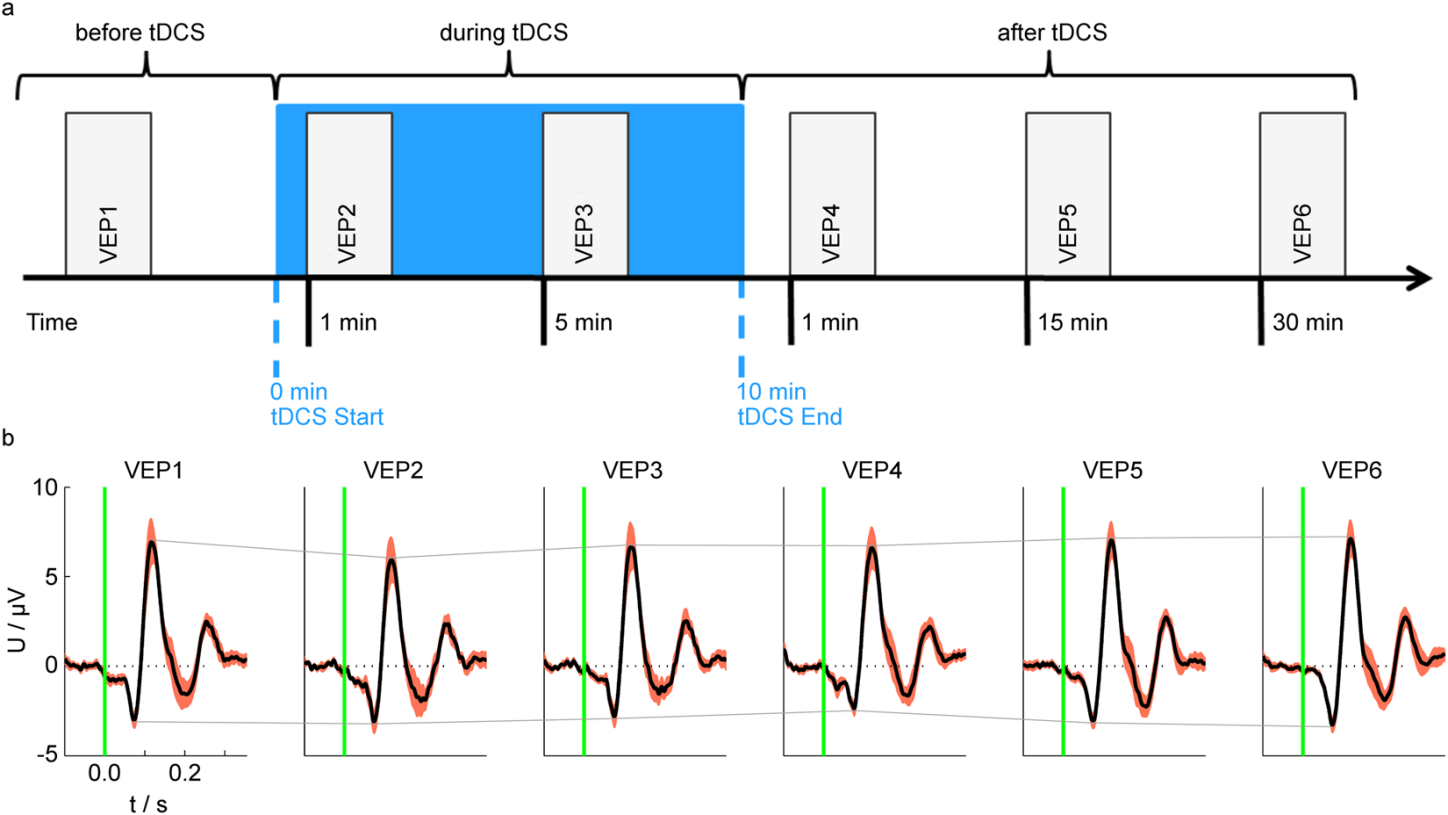
Grand averages indicate the amplitude reduction of the VEP components during and after tDCS. (**a**) Experimental procedure: the baseline session is indicated by VEP1. When tDCS was started, two VEP sessions were performed: VEP2 and VEP3. TDCS was applied for 10 minutes (blue area), followed by three additional post-stimulation sessions: VEP4, VEP5 and VEP6. (**b**) Grand average VEP (black) and standard error of the mean (red area) of all volunteers for all sessions in channel PO2. (The signals for the other channels exhibited similar characteristics.) The connecting solid lines between the amplitude peaks of the N75 and P100 components indicate the stimulation effects. | tDCS – transcranial direct current stimulation; VEP – visual evoked potential.

The objective of analysing the after-effects of stimulation was to compare the results obtained with the bifunctional cap to the results obtained with conventional TES–EEG equipment. The experimental paradigm was derived from Antal *et al*.^36^. The procedure was divided into six VEP sessions: before tDCS (VEP1: approximately 10 min before tDCS start), 1 min and 5 min after tDCS start (VEP2 and VEP3, respectively), and 1 min, 15 min and 30 min after tDCS end (VEP4, VEP5 and VEP6, respectively). The after-effects of cathodal tDCS on VEP components were evaluated by comparing VEP1, VEP4, VEP5 and VEP6 sessions. Figure 1f depicts the definition of the investigated VEP components: N75, P100 and N135. Visualizations of the experimental procedure and the corresponding grand average traces of one channel are presented in Fig. 2a,b.

**Figure 3:**
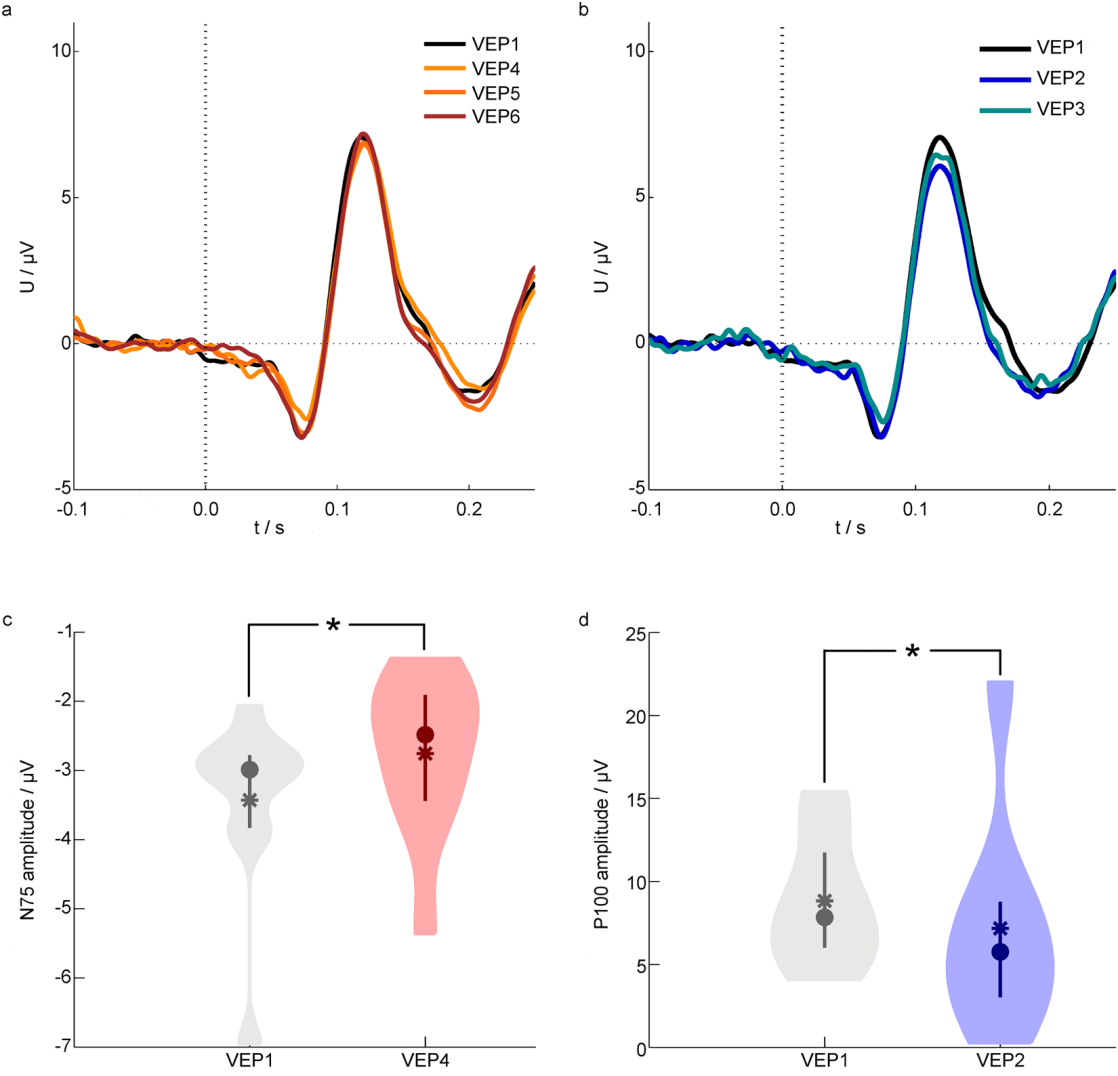
Grand average and statistical characteristics of the VEPs. (**a**) Grand average VEPs of all volunteers before and after tDCS in channel PO2. (**b**) Grand average VEPs of all volunteers before and during tDCS in channel PO2. (**c**–**d**) Violin plots of the amplitudes of the N75 (**c**) and P100 (**d**) components. Statistical characteristics are depicted by the mean (squares), median (circles), interquartile range (vertical lines) and the distribution of the data smoothed based on a normal kernel density estimation (coloured areas). Statistically significant differences are indicated by *. | tDCS – transcranial direct current stimulation; VEP – visual evoked potential.

To investigate the effect of tDCS on the magnitude of the VEP components, we analysed the peak amplitudes. In relation to the baseline (VEP1), the mean amplitudes of the N75 component were decreased by 12.6% in channel PO1, 19.6% in PO2 and 27.4% in POz for VEP4. The N75 amplitude was reduced significantly directly after tDCS (VEP4) compared to baseline (VEP1) (Wilcoxon rank sum test with Bonferroni corrected significance level p < 0.033, Supplementary Table 2). These findings indicate that cathodal tDCS over the visual cortex modulates the magnitude of the early VEP component, as presented in Fig. 3a (grand average trace) and Fig. 3c (statistical measures).

Further, our aim was to investigate the effect of tDCS on the temporal VEP characteristics by analysing the latency of the VEP components.

Directly after tDCS (VEP4), the P100 latency in channel POz was increased by 4.3% compared with baseline (VEP1). The P100 latencies in VEP5 and VEP6 were increased by 3.4% and 1.7% compared with baseline (VEP1).

This result might indicate that cathodal tDCS modulates the timing of cortical excitation.

Taken together, these results are in line with previously reported tDCS effects on VEPs and verify a reliable TES and EEG functionality of the novel cap.

### Direct-effects of tDCS

To evaluate the performance of the bifunctional cap at simultaneous TES–EEG experiments, we investigated the VEP sessions during tDCS. To identify these direct-effects (sometimes also called dynamic effects) of cathodal tDCS on VEP components, we evaluated the VEP parameters from sessions before versus during tDCS (VEP1 versus VEP2 and VEP3, respectively).

The signal quality of the VEP sessions which were acquired simultaneously to the tDCS, was similar to the ones without stimulation, as Fig. 1f exhibits.

To investigate the effect of concurrent tDCS on the magnitude of VEP components, we analysed the peak amplitudes.

During stimulation, the amplitude of the P100 component was decreased in all channels, as shown in Fig. 3b. Specifically, in relation to the baseline (VEP1), the mean amplitudes of the P100 component were decreased by 25.2% in channel PO1, 30.9% in P2 and 20.0% in POz for VEP2 as well as 29.9% in channel PO1, 25.3% in P2 and 18.5% in POz for VEP3, respectively. Statistical analysis (Wilcoxon rank sum test with Bonferroni-corrected significance level of p < 0.033, Supplementary Table 3) revealed a significant decrease of the P100 amplitude during tDCS (VEP2) compared with the baseline session VEP1, see Fig. 3d for statistical measures.

Exploring further VEP parameters, we found the peak-to-peak amplitudes for N75–P100 and P100–N135 were also modulated during tDCS in all channels. In both sessions during tDCS (VEP2 and VEP3), the peak-to-peak amplitude for N75–P100 in channel PO2 was decreased compared with the baseline session VEP1. During tDCS, the peak-to-peak amplitude for P100–N135 in channel PO2 in sessions VEP2 and VEP3 was decreased compared with the baseline session VEP1. These findings indicate that cathodal tDCS over the visual cortex modulates the magnitude of the P100 component directly during the course of stimulation.

Exploring the direct-effects of tDCS on the temporal VEP characteristics, we analysed the latencies of the VEP components. The P100 latency in channel PO2 was increased by 3.4% during tDCS (VEP3) compared with the baseline session VEP1. This might suggest that cathodal tDCS over the visual cortex modulates also the latency of the P100 component during the course of stimulation.

Taken together, the results provide new insights, how magnitude and latency of VEP components might be affected during stimulation.

As a decreased VEP magnitude might be linked to a reduction of activity, and an increased latency might be related to a prolonged reaction time, those findings indicate that cathodal TES reduces the excitability of the targeted cortical area.

## Discussion

We describe here for the first time the development of a flexible cap with integrated textile stimulation electrodes for TES and dry EEG recording electrodes, which enables simultaneous TES and EEG applications. We successfully verified the cap’s functionality by investigating the after-effect of tDCS on VEPs, and additionally observed novel direct stimulation effects during the course of tDCS. The functionality and handling of the bifunctional cap suggested that this new tool can overcome the problems and limitations of conventional TES–EEG systems.

During its application in the presented study, the bifunctional cap offered the following benefits. First, the integration of textile stimulation electrodes into a cap ensured a reproducible placement of the stimulation electrodes relative to each other, which is difficult to obtain in conventional TES due to the separate placement of the single electrodes with rubber straps^11^. Second, the flexible fabric facilitated optimal fitting to individual head shapes. Consequently, the full electrode area effectively contributed to the stimulation without partial displacements due to bending or protruding of some electrodes or incomplete contact through the hair layer as experienced in most conventional TES applications^49^. These two aspects can reduce the time burden for patients and volunteers and allow the use of the cap without a trained medical staff. Third, the elimination of the need for skin preparation and electrolyte gel reduces the risk of skin irritations and hair loss, which constitutes an advantage of our cap over other caps^40^ using gel-based Ag/AgCl electrodes for TES. A fourth benefit of the textile cap stimulation electrodes is that the stimulation electrode can be designed individually and thus areas and shapes are not restricted to the size of Ag/AgCl electrodes. This reduces the risk of exceeding the current density limit^10^ with too small electrode areas. Fifth, dry EEG electrodes do not limit the measurement duration, as it is the case with gel-based EEG electrodes due to gel dehydration^50^. Moreover, the use of abrasive gel and conductive paste for wet electrodes poses an inconvenience for the participant, because the preparation can be slightly harmful and the sticky and dirty hair and scalp need to be washed after the experiment^50^. In accordance with Fiedler *et al*.^46^, we found that dry multipin EEG electrodes passed through the hair and enabled a stable skin contact. Participants found that wearing of dry EEG electrodes was comfortable and reported no adverse skin sensations. Furthermore, the use of a cap with integrated dry multipin EEG electrodes can reduce the preparation time by 86% compared to gel-based Ag/AgCl EEG caps^46^.

The verification of our new textile stimulation electrodes showed already at the beginning of stimulation sufficiently low impedances between the anode and cathode, which further decreased over the course of stimulation. The textile stimulation electrodes exhibited an impedance behaviour like conventional TES electrodes (own unpublished data). The silicone frame successfully prevented diffusion of the saline solution and ensured stimulation stability over the duration of the experiments. These factors are important for the stability^11,49^ of the stimulation at the intended cortical area.

The performance of the textile stimulation electrodes did not degrade after 100 machine laundering cycles using the ‘delicate’ setting of the machine. While the cap was washed without the dry EEG electrodes for this purpose, which was not a general requirement, separate hand washing tests with a 64 channel dry electrode cap revealed no degradation on the EEG electrodes (observations made during another study^46^). Unlike hand washing, machine laundering guarantees a defined cleaning process. This is especially important for home use, where considerable cleaning operator variability is expected. Consequently, the possibility to clean the cap in a washing machine broadens the application potential for therapy and home use.

However, potential for optimization of this cap still exists. First, simultaneous EEG recording and tDCS application require a matching of the EEG amplifier properties (primarily dynamic range)^51^ with the tDCS stimulation (primarily low interfacial impedance between the stimulation electrode and the skin surface^49^). The amplifier used in this study requires an interfacial impedance between the anode and cathode of less than 5 kΩ, as recommended by the manufacturer. In some volunteers, this recommendation of was not achievable. The reason for the slightly increased impedances might be a weak contact pressure in certain areas of the stimulation electrode due to the curvature of the head. Consequently, we plan to investigate the contact pressure of the textile stimulation electrodes in the bifunctional cap in the future. Further optimization concerning the dry EEG electrodes is necessary, as they have a slightly increased channel drop-out rate (i.e. the percentage of channels failing to record EEG) compared with standard wet EEG electrodes as well as a higher susceptibility to movement artefacts, as discussed in Fiedler *et al*.^46^. The dry EEG electrodes also require sufficient contact pressure, which was estimated only for single electrodes to be 3–4 N^47^. Consequently, we plan to investigate the distribution of contact pressures for multiple dry electrodes in a bifunctional cap. A general limitation of all combined TES–EEG experiments is that no EEG electrodes can be placed at the area of the stimulation electrode, which is also true for our bifunctional cap. Another principal limitation of our and other caps is that extra-cephalic electrodes must be attached manually.

To verify the cap’s functionality, our aim was to reproduce a well-known TES effect on an EEG outcome measure, which was initially investigated by using conventional TES–EEG equipment. The study of the tDCS after-effect on VEPs reported by Antal *et al*.^36^ served as a basis, where they used rubber electrodes in water-soaked sponges for the application of TES and conventional wet Ag/AgCl electrodes for EEG recording. This setup represents a typical, conventional TES–EEG setup, which was also used in further studies^37,38^ investigating tDCS effects on VEPs.

The decrease of the N75 amplitude of 22.6% compared with the baseline before tDCS is in line with the previously reported effect of a significant decrease of the N75 amplitude after cathodal tDCS^36^. In accordance with Antal *et al*.^36^, we observed no significant difference in the P100 amplitude between the baseline (VEP1) and directly after tDCS (VEP4). Our experimental setup slightly differed from the one of Antal and colleagues^36^ concerning VEP generation (pattern-reversal versus pattern onset-offset for Antal *et al*.; mean luminance of 173.5 cd/m² versus 60 cd/m² for Antal *et al*.) due to our facilities. While Antal and colleagues^36^ also placed the stimulation electrode on Oz, their EEG electrodes were positioned at locations (O1, O2 and Oz) slightly different from ours. Despite these slight experimental differences, our findings are in line with Antal and colleagues^36^, indicating the robustness of the chosen paradigm and a successful verification of both functionalities of our novel cap.

When we explored VEPs simultaneously with TES, we identified indications for previously unreported tDCS effects. First, the amplitude of the P100 component was decreased significantly during cathodal tDCS. These results contradict those of Accornero *et al*.^37^, who observed a reciprocal effect of anodal and cathodal tDCS on the amplitude of the P100 component during and after the stimulation, also using pattern-reversal stimulation. Second, the P100 latency showed a trend to increase during and directly after the stimulation, and decreased in the following post-tDCS sessions. Our findings concerning the increase of the P100 latency during and after cathodal tDCS might indicate a stimulation effect on the P100 latency. In comparison, Accornero *et al*.^37^ reported no significant effect of cathodal tDCS on the P100 latency. A reason for these contradicting findings could be the different positions of the anodal stimulation electrode. Accornero *et al*.^37^ placed the cathode 1 cm above the inion and the anode over the anterior neck base or over C7, which resulted in a different electric field and direction of stimulation current compared with the montage used by Antal *et al*.^36^ and in our study. Further, the paradigm of the study by Accornero and colleagues differed from the recommendations of the International Society for Clinical Electrophysiology of Vision (ISCEV)^52^ regarding pattern repetition rate (2 Hz ± 20%), number of trials (90 trials), cut-off frequency of the bandpass filter (2 Hz) and position of the reference electrode for VEP recording (Cz). Third, we report here for the first time a decrease of the N75–P100 and P100–N135 peak-to-peak amplitudes during cathodal tDCS, which is due to the direct influence of the change of the P100 amplitude on these peak-to-peak amplitudes.

Zaehle *et al*.^53^ reported an effect of tDCS on event-related potential latencies in a visual working memory task and on the signal power in EEG frequency bands after tDCS. Strigaro *et al*.^38^ observed significant changes of the P2 latency and the amplitude of the late response of flash VEPs during and after anodal tDCS. Although the paradigms used in these two studies are different from our paradigm, we observed similar effects on latencies, which indicates a general influence of tDCS at the occipital area on the visual system.

The after-effects of tDCS on VEP components observed in the present study demonstrate variability over the analysed EEG channels. The after-effect of tDCS on the N75 amplitude was most pronounced in channel PO2. However, the direct-effect on the P100 amplitude was significant in all channels. As described by Baseler *et al*.^54^, the form and magnitude of a VEP signal strongly depends on the stimulus size, location and the individual cortical anatomy, which could affect the reported tDCS effects. To further investigate this variability of after-effects of tDCS on VEP components over the EEG channels, a quantitative TES–EEG mapping study with a large number of EEG channels is required. Such a study could also reveal EEG mapping differences and consequently differences in the localization of the underlying sources in the brain. Based on the found variability over the EEG channels, one would expect a different source configuration contributing the VEP components with and without TES.

The main direct stimulation effect reported here was statistically significant. However, the small sample sizes of 6 and 8 are a weak point of this analysis. Therefore, the novel tDCS effects should be reproduced with a larger sample size to confirm these findings. The lack of a sham condition additionally limits the newly observed direct stimulation effects. For evaluating the observed modulation effects on VEPs, a sham-controlled study including both anodal and cathodal tDCS is needed.

In conclusion, our study provides evidence for the functionality of the new bifunctional cap, which offers an attractive alternative to conventional TES–EEG systems. The cap can improve of the research practice for studies that use the combination of TES and EEG to investigate the mechanisms of TES from an electrophysiological perspective. By using a flexible cap with pre-defined, integrated stimulation and EEG electrodes, studies with repetitive applications or large numbers of participants could benefit from this new tool, especially regarding reproducibility and preparation time. In clinical practice, the bifunctional cap can improve monitoring of non-invasive brain stimulation. In addition, concurrent monitoring of TES outcome and adapting of TES parameters (closed-loop approach) is another broad field of application for this cap. TES has great therapeutic potential in the home care sector. Therefore, a bifunctional cap that applies the TES for therapy and monitors the course of stimulation by using a few EEG electrodes constitutes an optimal tool for this prospective scenario.

## Methods

### Subjects

Ten healthy (mean age 24.8 ± 2.5 years, 2 women) volunteers participated in the study. All volunteers were asked about potential contraindications^55^ and provided written informed consent. Concerning their visual function, only volunteers without or corrected refractive errors were included. The study protocol complied with the Declaration of Helsinki and was approved by the Ethics commission at the medical faculty of the Friedrich-Schiller-University Jena, Germany.

### Visual stimuli

Visual stimuli were created with eevoke, version 2.2 (Advanced Neuro Technology B.V., Enschede, The Netherlands) and presented on a LCD display MYRICA V30-1 (Fujitsu Siemens Computers GmbH, Augsburg, Germany), which measured 65 cm × 39 cm. The volunteers were seated in front of the monitor using a headrest, with their eyes 50 cm from the screen, resulting in a field of view of 66.0 degrees × 42.6 degrees. Checkerboard units measured 3.7 × 2.8 cm (1001 pixels × 751 pixels at 72 dpi) and a red cross in the center of the display served for visual fixation. The room was darkened. In accordance with the ISCEV^52^ recommendations, a black and white checkerboard pattern reversal was presented 300 times. The duration of 455 ms per pattern corresponded to a pattern repetition frequency of 1.1 Hz. A high pattern contrast was used (Michelson contrast of 98%) with a mean luminance of 173.5 cd/m².

### VEP recording

Visual evoked potentials were recorded on seven channels (POz, PO1, PO2, PO5, PO6, PO9, PO10) according to the international 10/10 system as depicted in Fig. 1c. Dry EEG electrodes consisting of a common baseplate with 30 pins made out of polyurethane and coated with Ag/AgCl^46^ were used for recording (Fig. 1e). The dry multipin electrodes were integrated in a flexible cap with textile stimulation electrodes. Conventional sintered Ag/AgCl ring electrodes that served as a reference and a ground electrode were positioned at Fz and AFz, using Ten-20 conductive EEG paste (Weaver and Company, Aurora, USA), respectively. A NEURO PRAX MR system (neuroConn GmbH, Ilmenau, Germany) with a DC-coupled EEG amplifier was used for EEG recordings. With a dynamic range of ±184 mV and 24-bit digitization, high-quality EEG recordings were provided during TES. VEPs were recorded with a sampling rate of 4000 Hz.

### Transcranial direct current stimulation

The tDCS was applied using the DC-STIMULATOR PLUS (neuroConn GmbH, Ilmenau, Germany). Electrodes were placed according to the international 10–20-system at Oz for cathodal stimulation of the visual cortex and at Cz as the anodal return electrode (Fig. 1a–c). Textile silver electrodes^48^ (Fig. 1d) were used for tDCS and were also integrated into the flexible cap (warmx GmbH, Apolda, Germany). A saline-soaked sponge was placed in a textile pocket on the outside of the cap to ensure low-impedance electrical contact between the stimulation electrode and the scalp. The size of the textile electrodes measured 4.5 cm × 3.5 cm over Oz and 4.5 cm × 4.0 cm over Cz when the cap was positioned on a head model with a head circumference of 58 cm. The electrical connection between the electrode cables (snap leads) and the stimulation electrode was established by attaching a snap fastener to the conductive part of the fabric. A silicone frame was integrated around the textile electrodes to prevent diffusion of saline solution into the textile outside of the electrode area (Fig. 1b).

The stimulation intensity was linearly increased from 0 to 1 mA for 5 s in the beginning of stimulation to prevent transient sensations. After this fade-in phase, the maximum current of 1.0 mA was applied for 10 min and linearly decreased for 5 s afterwards for same reasons. The manufacturer’s recommendation for a stable combination of EEG and tDCS with the utilized equipment included an interface impedance between the stimulation electrode and the skin of less than 5 kΩ to prevent saturation of the EEG amplifier during tDCS. Consequently, we attempted to reach electrode–skin impedances below 5 kΩ, which was achieved in 5 out of 10 volunteers.

We tested the stimulation electrodes by assessing electrode–skin impedance and diffusion behaviour after 50 and 100 machine launderings using Spee liquid (Henkel AG & Co. KGaA, Düsseldorf, Germany) in the delicate setting (W1, Miele & Cie. KG, Gütersloh, Germany). Electrode–skin interface impedance of the textile cathode was measured against a standard Ag/AgCl reference electrode applied to the forehead with Ten-20 conductive EEG paste (Weaver and Company, Aurora, USA). The diffusion behaviour was assessed at 4 positions in the textile surrounding the silicone frame. These positions were roughly at the 10–10 positions of POz, PO5, PO6 and Iz. At these four positions, the nonconducting textile was connected with alligator clips. Impedance measurements were performed between the standard Ag/AgCl reference electrode at the forehead and the four positions around the silicone frame. In cases where the silicone barrier performed according to specifications, no diffusion of the saline solution should occur, and these positions should be non-conducting, and high impedance values should occur. In the undesired cases of saline solution diffusion to the outside of the silicone frame, low impedance values should occur. Both electrode–skin impedance and diffusion behaviour impedances were measured five times in 100 samples at 10 Hz with a 2,000 kΩ cutoff using the HP 41492A LF Impedance Analyzer (Hewlett-Packard Company, Palo Alto, USA). After an initial settling time of 10 min, we measured 100 samples at each position interleaved (textile cathode and four positions around the silicone frame) and repeated this procedure five times. Consequently, all impedance measurements lasted for 30 min.

Further, the flexibility of the textile material of the cap material was tested by means of tensile tests using a compression-tension test equipment (Z005, Zwick GmbH & Co. KG, Ulm, Germany). Tension was averaged over three samples separately along and orthogonal to the knitting direction. We used a force limit of 20 N as the expected tension force during cap application.

### Experimental procedures

First, the cap was positioned on the volunteer’s head. After placing the reference and ground electrodes, the volunteer’s head was positioned in a headrest for visual stimulation. Six VEP recordings were performed: one before, two during and three after tDCS (Fig. 2). VEP1 represents the baseline session. Next, the saline-soaked sponge pads for tDCS were inserted into corresponding pockets of the cap, and the stimulation was initiated. During tDCS, two VEP sessions were performed at 1 min (VEP2) and 5 min (VEP3) after the tDCS start to analyse the direct tDCS effects. At the end of tDCS, the sponge pads were removed from the cap, and post-tDCS VEP sessions were performed at 1 min (VEP4), 15 min (VEP5) and 30 min (VEP6) to analyse the after-effects. All sessions were performed by the same operator.

### Data analysis

Data processing was performed with MATLAB, version 2011b (The Mathworks, Inc., Natick, USA). All active channels were referenced to Fz and offset-corrected. Filters were applied with the following specifications: comb filter at 50 Hz and harmonics (Butterworth, order: 80) and bandpass filter from 1 Hz to 100 Hz (Butterworth, order: 10) according to the recommendations of the ISCEV^52^. Manual artefact identification was performed and corresponding trials were excluded from further analysis. The number of trials with artefacts across all volunteers and sessions varied between 75 and 10. Consequently, we used 225 trials for all volunteers as it represents the minimum number of artefact-free trials for each session. Baseline correction was applied using the mean of each trial -100 ms–0 ms before the stimulus, eliminating signal offsets. Averaging was performed with 225 trials for each session and each volunteer. The grand average of the recordings of all volunteers was calculated for each channel and each session. Amplitudes (peak maximum), latencies (time between visual stimulus and peak maximum) and peak-to-peak amplitudes of the N75, P100 and N135 components were calculated (Fig. 3) and visually inspected (Fig. 2). Based on the visual inspection, the most prominent changes in the VEP morphologies have been selected for statistical analysis.

### Statistical analysis

Statistical analysis was performed using IBM SPSS Statistics, version 22 (IBM Corp., Armonk, USA). The Shapiro-Wilk test demonstrated that the data were not normally distributed, thus we selected nonparametric procedures for further analysis. Comparisons between the conditions before and after tDCS were performed for 10 volunteers. Comparisons between the intervals before and during tDCS were performed for 8 volunteers in channel PO2 and 6 volunteers in channels PO1 and POz. For the other volunteers, EEG signals during tDCS were rejected from the analysis for technical reasons. Wilcoxon rank sum tests for two connected samples (two groups of repeated measures) were performed with α = 0.1, appropriate for a proof of principle study with few participants. Statistical tests were performed for the three EEG channels (PO1, PO2, POz) separately. To control the family-wise error rate of these multiple comparisons for the correlated channels, we applied the Bonferroni correction.

The datasets generated during and/or analysed during the current study are available from the corresponding author on request.

## Electronic supplementary material

Supplementary Material
