# Supplementary material for: Novel bifunctional cap for simultaneous electroencephalography and transcranial electrical stimulation
Source: Sci Rep. 2018 May 8;8:7259. doi: 10.1038/s41598-018-25562-x (PMC5940899; doi:10.1038/s41598-018-25562-x)
Supplement: Supplementary file 1 — Supplementary Material [file 41598_2018_25562_MOESM1_ESM.pdf]

Novel bifunctional cap for simultaneous electroencephalography and transcranial electrical stimulation

Sophia Wunder[1,2], Alexander Hunold[1], Patrique Fiedler[1], Falk Schlegelmilch[2], Klaus Schellhorn[2], Jens Haueisen[1,3]

[1] Institute of Biomedical Engineering and Informatics, Technische Universität Ilmenau, 98693 Ilmenau, Germany

[2] neuroConn GmbH, 98693 Ilmenau, Germany

[3] Department of Neurology, Biomagnetic Center, Jena University Hospital, 07747 Jena, Germany

Supplementary Material

Supplementary Table 1 | Results of impedance measurements between one Ag/AgCl reference electrode on the forehead and the textile stimulation electrode area and four position on the textile outside the diffusion barrier, respectively. This proposes a successful verification the cleaning procedure and integrity of the diffusion barrier.

	Impedance / kΩ between reference electrode and positions on the:					
	Inside of the stimulation area		Outside of the diffusion barrier			
	Mean	Standard deviation	Mean	Standard deviation	Maximum	Minimum
Before laundering	40.2	3.1	466.2	434.0	1300.0	108.7
After 50 laundering cycles	38.5	5.6	510.4	336.6	1300.0	110.0
After 100 laundering cycles	26.7	14.4	407.6	307.9	1290.0	84.7

Supplementary Table 2 | The results of statistical analysis between pre- and post-tDCS (VEP1 and VEP4) are listed for the amplitude of the analyzed VEP components in the EEG (PO1, PO2, POz).

	VEP component	p-value		
		PO1<br>n = 10	PO2<br>n = 10	POz<br>n = 10
Amplitude	N75	0.241	0.022 *	0.093

* Asymptotic significance of Wilcoxon rank sum test (p < 0.033 Bonferroni corrected)

Supplementary Table 3 | The results of statistical analysis between pre-tDCS and during tDCS (VEP1and VEP2) are listed for the amplitude of the analyzed VEP components in the EEG (PO1, PO2, POz).

	VEP component	p-value		
		PO1<br>n = 6	PO2<br>n = 8	POz<br>n = 6
Amplitude	P100	0.028 *	0.012 *	0.028 *

* Asymptotic significance of Wilcoxon rank sum test (p < 0.033 Bonferroni corrected)
